# Melatonin-mediated mitophagy protects against early brain injury after subarachnoid hemorrhage through inhibition of NLRP3 inflammasome activation

**DOI:** 10.1038/s41598-017-02679-z

**Published:** 2017-05-25

**Authors:** Shenglong Cao, Sudeep Shrestha, Jianru Li, Xiaobo Yu, Jingyin Chen, Feng Yan, Guangyu Ying, Chi Gu, Lin Wang, Gao Chen

**Affiliations:** Department of Neurosurgery, The Second Affiliated Hospital of Zhejiang University School of Medicine, Hangzhou, China

**Keywords:** Molecular biology, Stroke

## Abstract

The NLRP3 inflammasome is activated in the early period following subarachnoid hemorrhage(SAH), resulting in inflammatory responses. Recent studies have shown that activation of NLRP3 inflammasome is suppressed by autophagy, but the potential mechanism is unclear. In this study, we examined whether mitophagy was involved in the beneficial effect of melatonin and its relationship with NLRP3 inflammasome activation after SAH. In total, 130 adult-male SD rats were randomly divided into four groups: sham group, SAH + vehicle group, SAH + melatonin group, and SAH + 3-methyladenine (3-MA) + melatonin group. Brain samples were used for brain water content analysis, ROS assay, Western blot, immunohistochemistry and transmission electron microscopy. The results showed that melatonin treatment markedly increased the expression of both autophagy markers(LC3-II/LC3-I and Atg 5), and mitophagy markers(Parkin and PINK-1) following SAH induction. Additionally, melatonin treatment attenuated pathological changes in mitochondria and reduced ROS generation, which are closely related to NLRP3 inflammasome activation. Consequently, melatonin-mediated upregulation of proteins associated with mitophagy inhibited NLRP3 inflammasome activation and significantly reduced pro-inflammatory cytokine levels after SAH. Conversely, 3-MA, an autophagy inhibitor, reversed these beneficial effects of melatonin on mitophagy and the NLRP3 inflammasome. These results suggest that mitophagy-associated NLRP3 inflammasome inhibition by melatonin is neuroprotective against early brain injury post-SAH in rats.

## Introduction

Subarachnoid hemorrhage (SAH) is a devastating form of stroke with high mortality and morbidity^1^. Recent studies indicate that early brain injury (EBI) plays a critical role in the poor outcomes of patients with SAH. Over the last decade, growing evidence has demonstrated that neuroinflammation contributes to injury progression in the early stage following SAH^2–4^. The NLRP3 inflammasome is assumed to mediate post-stroke brain injury^5–7^. Upon NLRP3 inflammasome activation, the maturation and secretion of IL-1β and IL-18 initiates the pro-inflammatory cell death pathway known as pyrotosis^8^. Pharmacologic inhibition or knock down of the NLRP3 inflammasome reduced mature IL-1β and IL-18 secretion and exerted a neuroprotective effect on post-SAH EBI in a rat model^7^.

Reactive oxygen species (ROS) is reported to induce NLRP3 inflammasome activation^9^. Damaged mitochondria in the injured brain are the main site of intracellular ROS generation^10^. Studies have demonstrated that inhibition of ROS generation and elimination of dysfunctional mitochondria are potential therapies for post-SAH EBI^11, 12^. Mitophagy, a selective form of autophagy that specifically removes damaged mitochondria, is critical for maintaining mitochondrial homeostasis and cellular survival^13^. When mitophagy is impaired, the accumulation of damaged mitochondria could result in the generation of mitochondrial ROS, which can pass through the plasma membrane^14^ and induce NLRP3 inflammasome activation. Thus, mitophagy is a potential therapeutic target for inflammasome-mediated cell death. Although the detailed mechanisms of mitophagy are still unknown, studies have demonstrated that mitophagy-meditated clearance of damaged mitochondria is related to the PINK1/Parkin pathway^15, 16^.

Melatonin is protective in EBI after SAH by enhancing autophagy, and by reducing oxidative stress, inflammation and apoptosis^17–21^. The protective mechanism of melatonin in SAH models remains unknown. To date, no study has investigated the influence of melatonin on mitophagy and its relationship with the NLRP3 inflammasome in SAH models. In the current study, we focused on the role of melatonin in the modulation of mitophagy and the relationship between these effects and NLRP3 inflammasome activation in EBI to improve the understanding of the neuroprotective effect of melatonin in SAH models.

## Results

### Melatonin attenuated brain edema and neurological dysfunction after SAH

**Figure 1:**
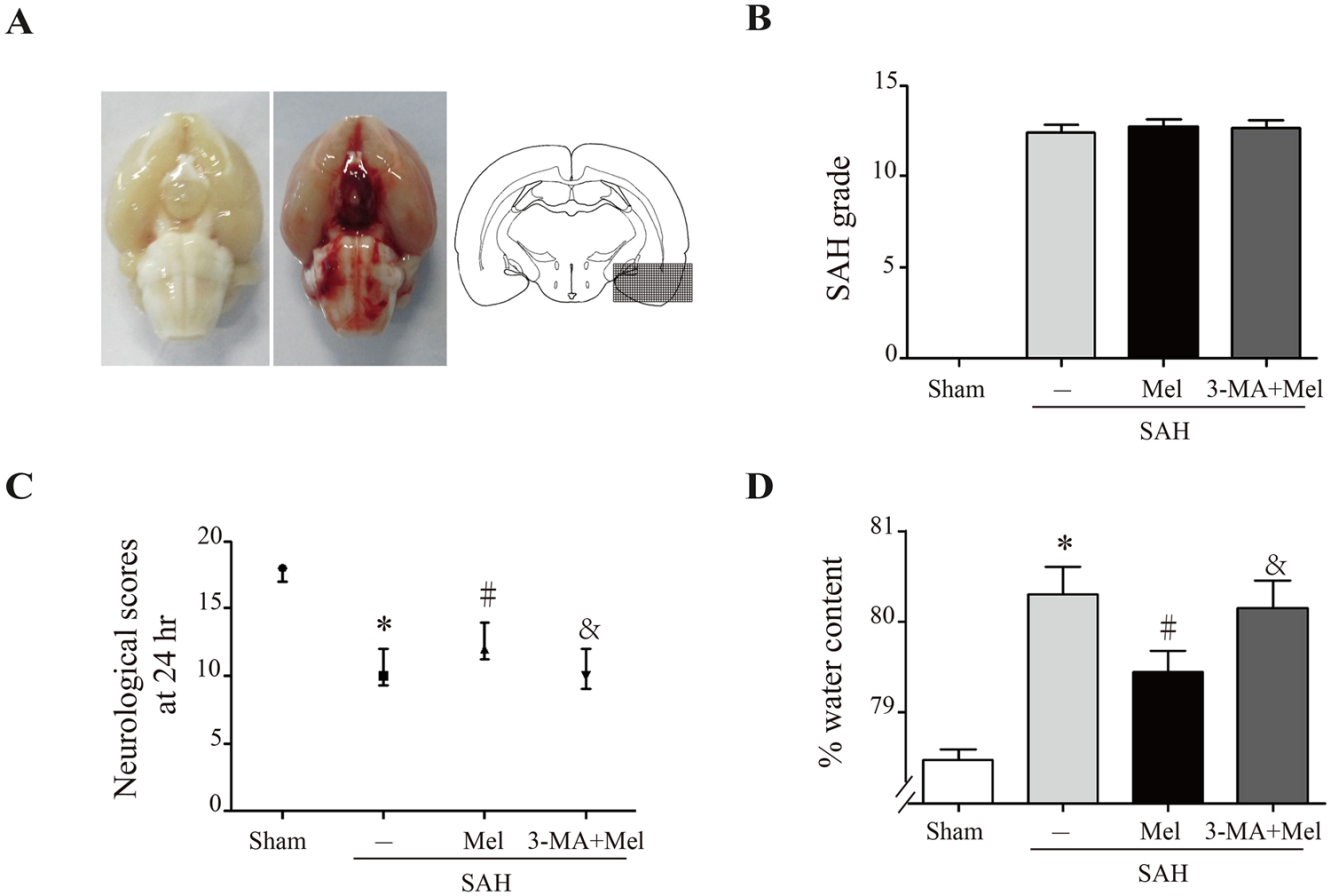
Effect of melatonin treatment on brain injury 24 h after SAH induction. (**A**) Representative brains from the sham and SAH groups, and the optimal region of brain section for immunochemistry. (**B**) The quantification of SAH severity, n = 24 per group. (**C**) The quantification of neurological scores. Values are presented as medians (interquartile range); n = 24 per group. (**D**) Brain water content measured by the wet-dry method; n = 6 per group. *P < 0.05 versus sham group, ^#^*P* < 0.05 versus SAH + vehicle group, and ^&^*P* < 0.05 versus SAH + Mel group.

All rats survived the sham operation. The mortality rate was 33.3% (n = 12/36) in the SAH + vehicle group, 25% (n = 8/32) in the SAH + Mel group and 36.8% (n = 14/38) in the SAH + 3-MA + Mel group. Representative brains from the sham and SAH groups are presented in Fig. 1A. The SAH grade of the sham group was 0, and SAH grades were not significantly different between the SAH + vehicle group, SAH + Mel group and SAH + 3-MA + Mel group (n = 24, Fig. 1B). Neurological deficits were evaluated using the scoring system of Garcia *et al*.^22^. Neurological scores were significantly lower in the SAH + vehicle group than in the sham group (n = 24, *P* < 0.05 SAH + vehicle vs sham, Fig. 1C). Melatonin treatment increased the neurological scores significantly, and the SAH + 3-MA + Mel group had a lower score than the SAH + Mel group (n = 24, *P* < 0.05 SAH + vehicle and SAH + 3-MA + Mel vs SAH + Mel, Fig. 1C).

Brain edema was quantified as changes in brain water content as measured by the dry-wet method^23^ and was analyzed 24 h after SAH induction. Whole-brain water content was significantly higher in the SAH + vehicle group than in the sham group. Rats treated with melatonin exhibited reduced brain edema compared with the SAH + vehicle group, while 3-MA pretreatment reversed these changes (n = 6, *P* < 0.05 SAH + vehicle and SAH + 3-MA + Mel vs SAH + Mel, Fig. 1D).

### Melatonin up-regulated mitophagy proteins and reduced ROS generation after SAH

**Figure 2:**
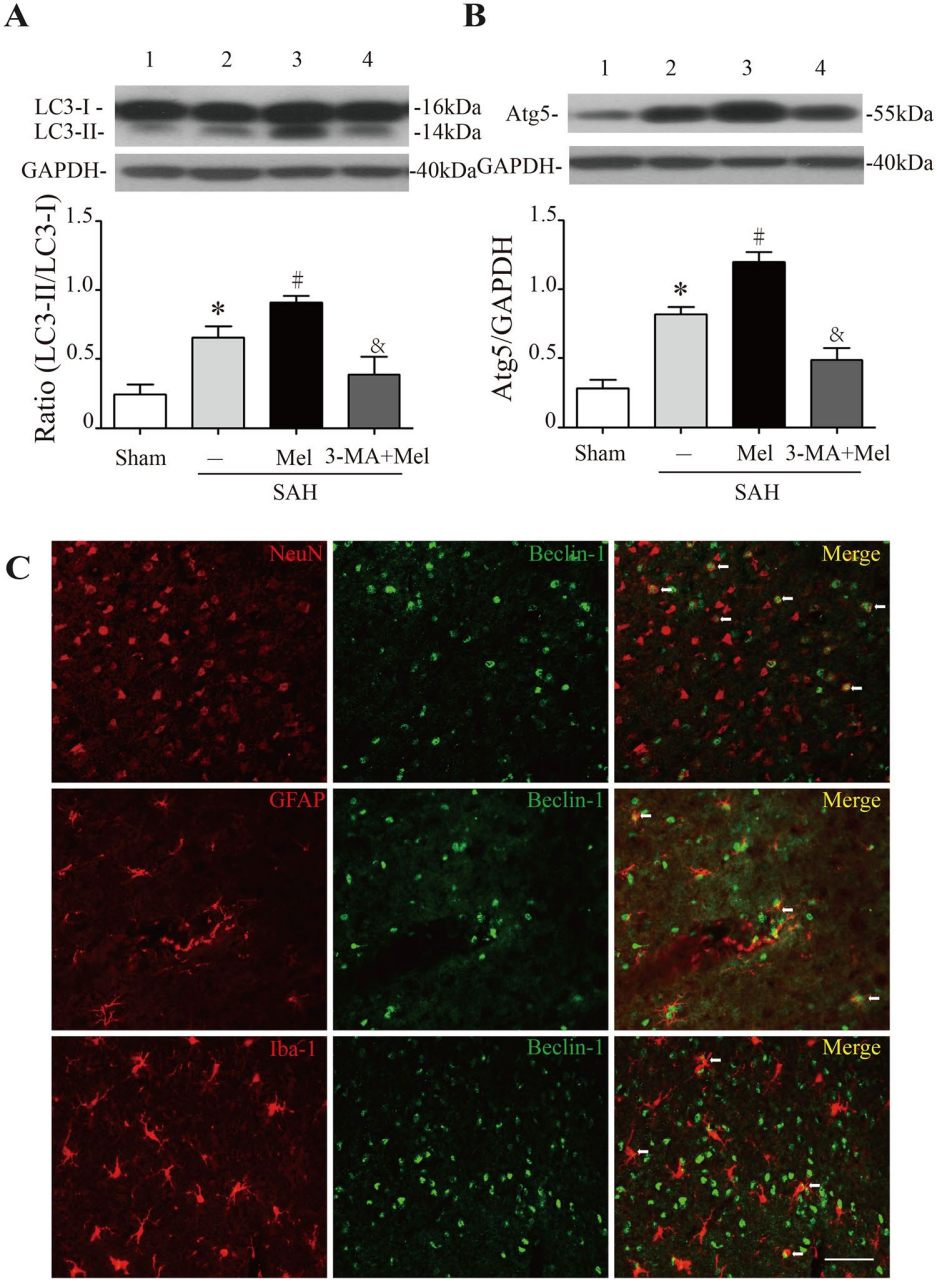
Effect of melatonin treatment on autophagy. (**A**) The Western blot relative band density of LC3; n = 6 per group. (**B**) The Western blot relative band density of Atg5; n = 6 per group. The cropped bands had been run under the same experimental conditions. (**C**) Representative micrographs showing double immunofluorescence labeling of Beclin-1 with NeuN (neuronal marker), GFAP (astrocyte marker) and Iba-1 (microglia marker) in the ipsilateral basal cortex at 24 h after SAH. White arrows indicated double-labeled cells. Scale bar = 50 μm. *P < 0.05 versus sham group, ^#^*P* < 0.05 versus SAH + vehicle group, and ^&^*P* < 0.05 versus SAH + Mel group.

To evaluate autophagy activation after melatonin treatment, we examined the expression levels of autophagy-related proteins, including LC3 and Atg 5. The conversion of LC3-I (a cytosolic form) to LC3-II (an active form) and Atg 5 expression levels were significantly increased in the melatonin-treated group compared with the SAH + vehicle group. The autophagy inhibitor 3-MA markedly reduced the ratio of LC3-II/LC3-I and the protein expression of Atg 5 (n = 6, *P* < 0.05 SAH + vehicle and SAH + 3-MA + Mel vs SAH + Mel, Fig. 2A,B). Double immunostaining showed that Beclin-1-positive cells were mainly expressed on NeuN-positive cells after SAH induction as well as on GFAP-positive cells and Iba-1-positive cells (Fig. 2C). These results indicated that autophagy-associated protein could be expressed in some cell types in the brain after SAH.

**Figure 3:**
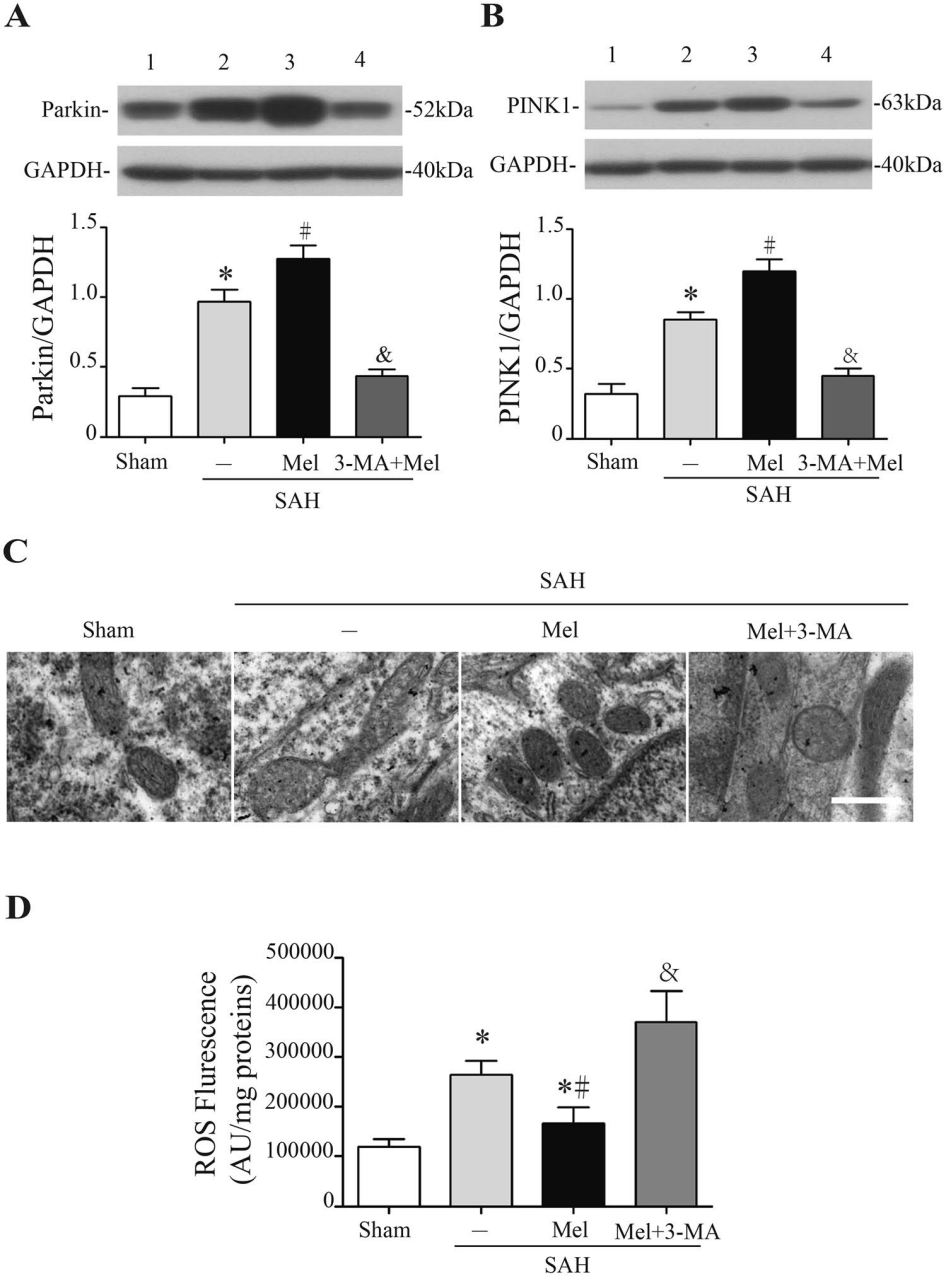
Effect of melatonin treatment on mitophagy and ROS generation. (**A**) The Western blot relative band density of Parkin; n = 6 per group. (**B**) The Western blot relative band density of PINK1; n = 6 per group. The cropped bands had been run under the same experimental conditions. (**C**) Ultrastructural changes in mitochondria in the ipsilateral basal cortex at 24 h after SAH induction. (**D**) The quantification of ROS levels in the ipsilateral basal cortex at 24 h after SAH; n = 6 per group. *P < 0.05 versus sham group, ^#^*P* < 0.05 versus SAH + vehicle group, and ^&^*P* < 0.05 versus SAH + Mel group.

Mitophagy, selective autophagy of mitochondria, mediates the clearance of damaged mitochondria by a pathway containing PINK1 and Parkin proteins^15, 16^. Western blot analysis showed that PINK1 and Parkin protein expression was increased at 24 h after SAH compared with the control, whereas melatonin treatment further up-regulated this expression (n = 6, *P* < 0.05 sham and SAH + Mel vs SAH + vehicle, Fig. 3A,B). Moreover, pretreatment with 3-MA inhibited the up-regulation of these proteins (n = 6, *P* < 0.05 SAH + 3-MA + Mel vs SAH + Mel, Fig. 3A,B). Since the upregulation of proteins associated with mitophagy occurred after melatonin treatment, we further investigated the ultrastructural phenotypes of the mitochondria. Under physiological conditions, mitochondria in the cortex of control rats appeared normal. In contrast, swollen mitochondria with cristae disarrangement and partial cristolysis were found in the cortex of SAH rats. Melatonin treatment attenuated morphological changes in the mitochondria, while 3-MA pretreatment reversed the beneficial effect of melatonin (Fig. 3C). Mitochondria are suspected to be the main site of intracellular ROS generation. ROS levels were significantly increased 24 h after SAH, and melatonin treatment markedly reduced the ROS content compared with vehicle-treated rats (n = 6, *P* < 0.05 sham and SAH + Mel vs SAH + vehicle, Fig. 3D). Inhibition of mitophagy by 3-MA exacerbated the generation of ROS after SAH (n = 6, *P* < 0.05 SAH + 3-MA + Mel vs SAH + Mel, Fig. 3D). These results suggested that treatment with melatonin increased mitophagy activity and reduced ROS generation after SAH induction.

### Melatonin inhibited NLRP3 inflammasome activation and attenuated pro-inflammatory cytokine secretion after SAH

**Figure 4:**
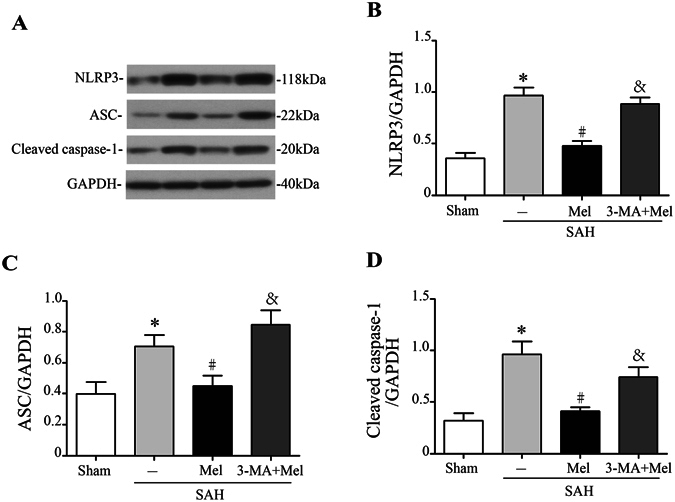
Melatonin treatment inhibits NLRP3 inflammasome activation. (**A**) Representative Western blot bands of NLRP3, ASC, and cleaved caspase-1. (**B**–**D**) The relative band densities of NLRP3, ASC, and cleaved caspase-1; n = 6 per group. The cropped bands had been run under the same experimental conditions. *P < 0.05 versus sham group, ^#^*P* < 0.05 versus SAH + vehicle group, and ^&^*P* < 0.05 versus SAH + Mel group.

Accumulation of ROS is a major trigger of NLRP3 inflammasome activation, which amplifies the inflammatory response and promotes pro-inflammatory cytokine secretion^9, 24, 25^. Western blot analysis showed that the expression of NLRP3, ASC and caspase-1 were low in the sham group, increased greatly after SAH induction, and the increase was suppressed by melatonin administration (n = 6, *P* < 0.05 sham and SAH + Mel vs SAH + vehicle, Fig. 4A–D). However, 3-MA administration reversed the effects of melatonin on the expression of these proteins (n = 6, *P* < 0.05 SAH + 3-MA + Mel vs SAH + Mel, Fig. 4A–D).

**Figure 5:**
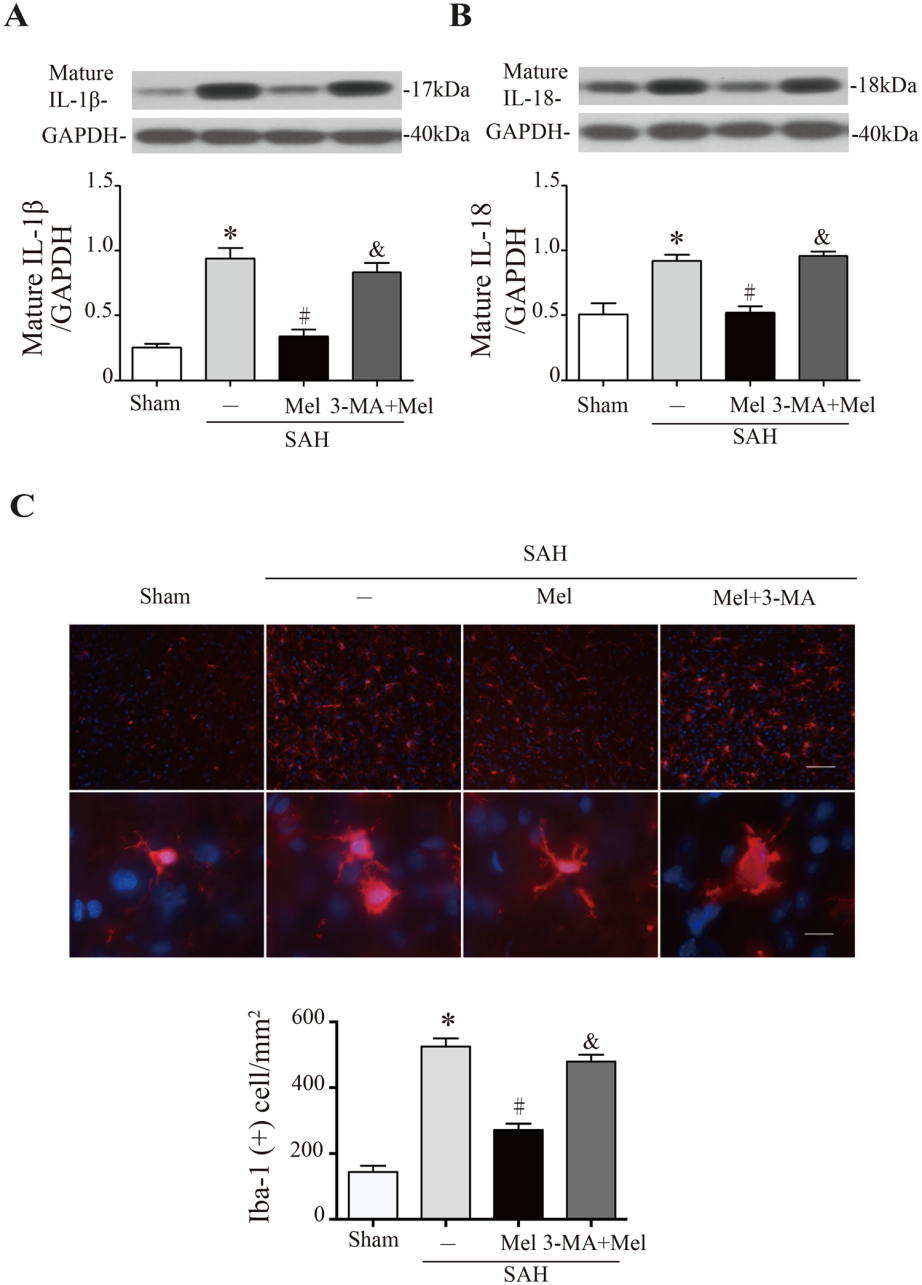
Melatonin treatment inhibits microglia activation and pro-inflammatory cytokine secretion. (**A**) The Western blot relative band density of IL-1β; n = 6 per group. (**B**) The Western blot relative band density of IL-18; n = 6 per group. The cropped bands had been run under the same experimental conditions. (**C**) Upper: representative micrographs showing microglial activation in the lesion area at 24 h after SAH, with immunofluorescence for Iba-1. Upper scale bar = 50 μm, lower scale bar = 10 μm. Lower: quantification of Iba-1-positive cells. *P < 0.05 versus sham group, ^#^*P* < 0.05 versus SAH + vehicle group, and ^&^*P* < 0.05 versus SAH + Mel group.

NLRP3 inflammasome activation leads to the secretion of pro-inflammatory cytokines, including IL-1β and IL-18. Western blot analysis showed that the expressions of IL-1β and IL-18 increased significantly at 24 h after SAH, whereas melatonin treatment markedly reduced the expressions of IL-1β and IL-18 compared with vehicle-treated rats (n = 6, *P* < 0.05 sham and SAH + Mel vs SAH + vehicle, Fig. 5A,B). Microglia are brain-resident macrophages that are activated in response to tissue injury. Microglial activation was obviously observed after SAH compared with sham controls, and melatonin significantly attenuated microglial activation compared with the SAH group 24 h after SAH (n = 6, *P* < 0.05 sham and SAH + Mel vs SAH + vehicle, Fig. 5C). Microglial activation was assessed by morphology. Loss of ramification and thickening of processes were observed after SAH compared to sham controls but were reversed significantly by melatonin treatment (Fig. 5C). Moreover, 3-MA inhibited the beneficial effects of melatonin on pro-inflammatory cytokine secretion and microglial activation (*P* < 0.05 SAH + 3-MA + Mel vs SAH + Mel, Fig. 5A–C).

### Melatonin reduced neuronal cell death after SAH

**Figure 6:**
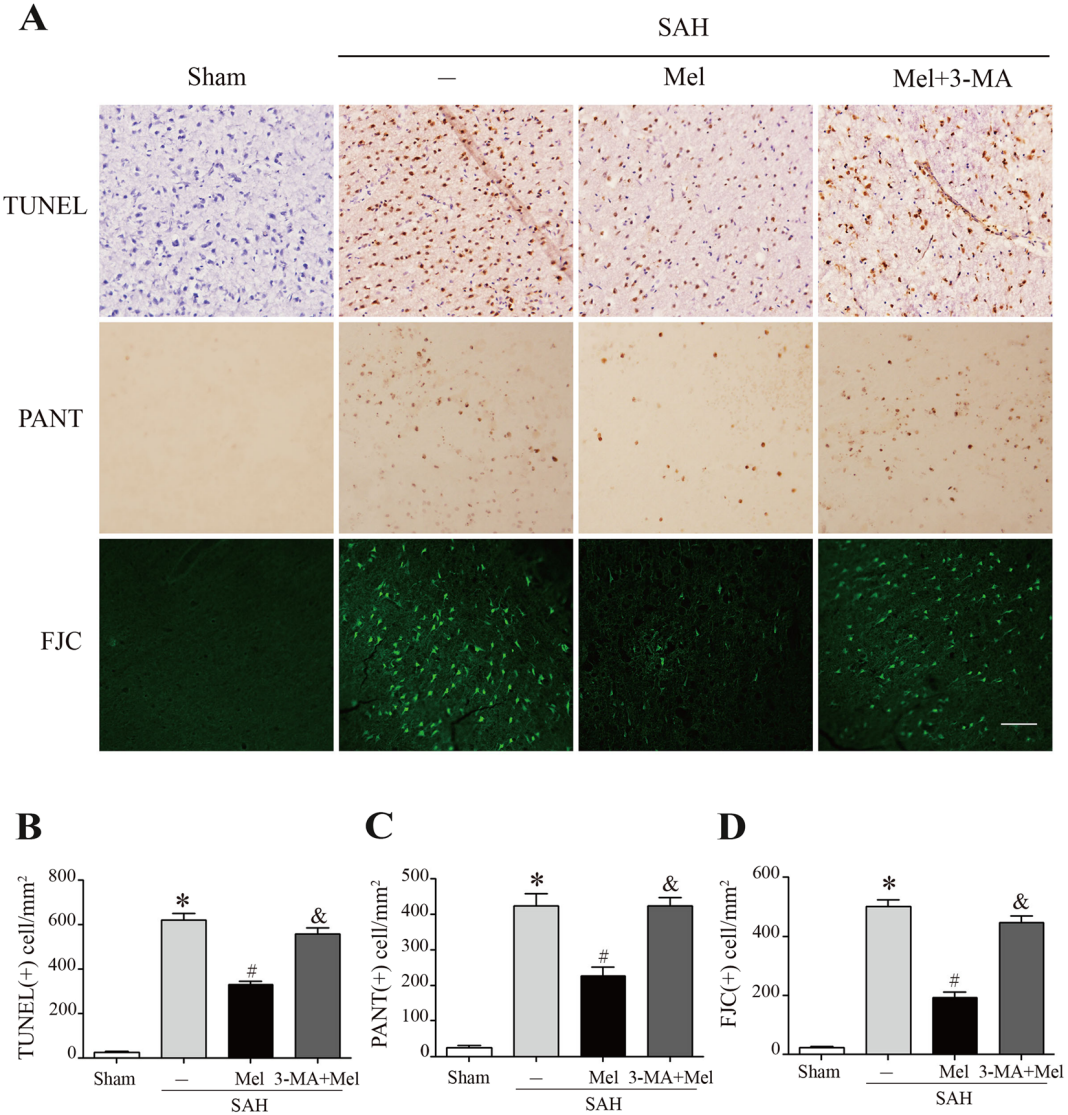
Effect of melatonin treatment on neuronal cell death. (**A**) Representative micrographs showing TUNEL, PANT and Fluoro-Jade C staining in the ipsilateral basal cortex at 24 h after SAH. Scale bar = 50 μm. (**B**–**D**) The quantification of TUNEL-positive cells, PANT-positive cells, and Fluoro-Jade C-positive cells. *P < 0.05 versus sham group, ^#^*P* < 0.05 versus SAH + vehicle group, and ^&^*P* < 0.05 versus SAH + Mel group.

Fluoro-Jade C staining was used for the detection of degenerating neurons, TUNEL staining was used to detect cells with double-stranded DNA damage, and PANT staining was used to detect cells with single-stranded DNA damage. Fluoro-Jade C-positive cells, PANT-positive cells and TUNEL-positive cells were rarely observed in the sham-operated rats, whereas the number of positive cells increased significantly in the SAH + vehicle group compared with the sham group. Melatonin treatment dramatically reduced the number of Fluoro-Jade C-positive cells, PANT-positive cells and TUNEL-positive cells. However, 3-MA pretreatment reversed the protective effects of melatonin on neuronal cell death (n = 6, *P* < 0.05 SAH + vehicle and SAH + 3-MA + Mel vs SAH + Mel, Fig. 6A–D).

## Discussion

**Figure 7:**
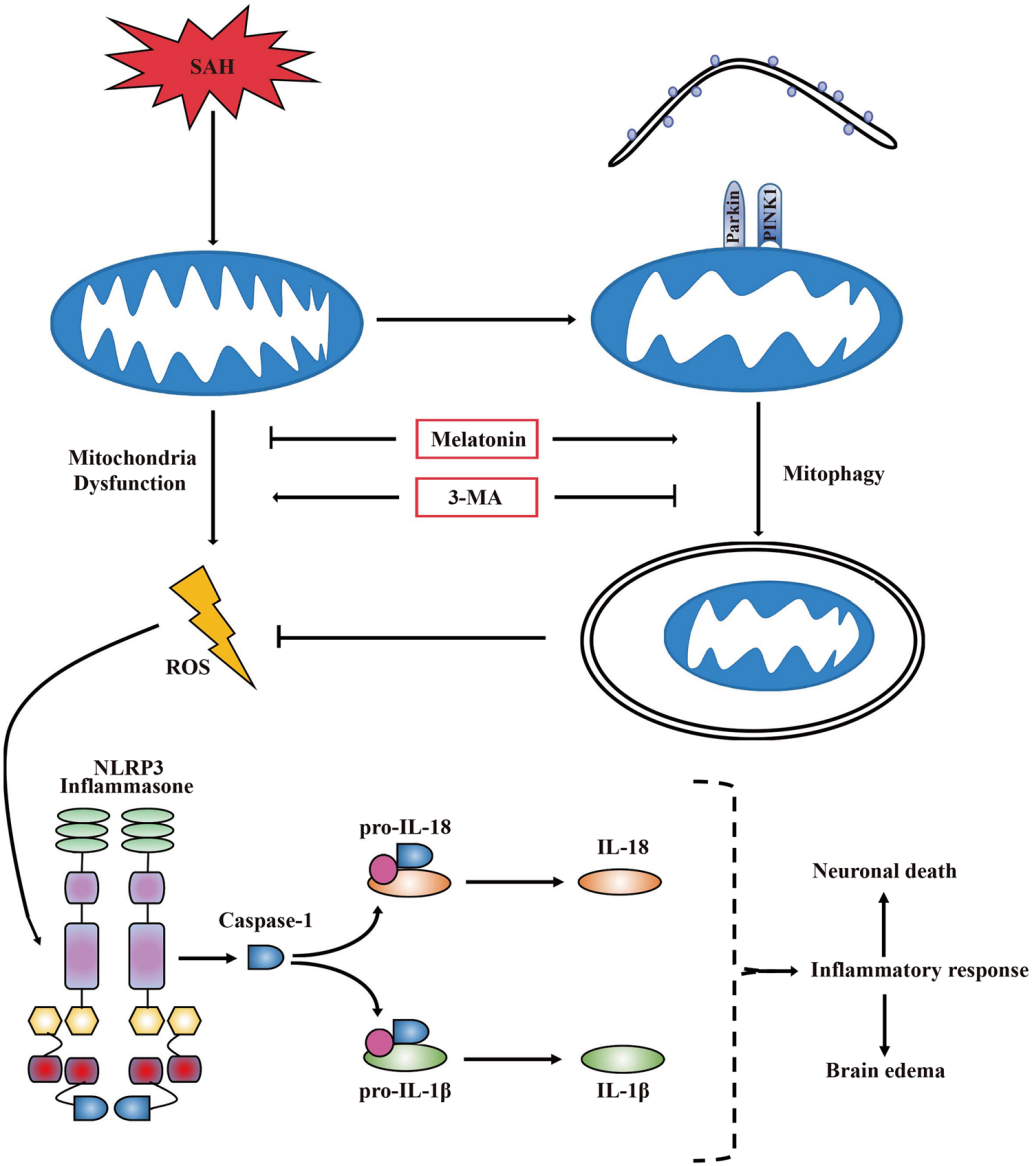
A potential process illustrating the effect of melatonin on mitophagy and the NLRP3 inflammasome following SAH. Briefly, SAH-induced damaged mitochondria released ROS and then activated the NLRP3 inflammmasome and secondary inflammatory responses. Treatment with melatonin upregulated mitophagy and reduced ROS generation, which resulted in positive feedback to inhibit the NLRP3-mediated inflammatory response. Mitophagy inhibitor 3-MA pretreatment reversed the beneficial effect of melatonin on the inhibition of the NLRP3-induced inflammatory response.

EBI refers to the immediate injury after SAH and is the primary cause of mortality and morbidity in patients with SAH. The treatment of EBI is a major target in the management of survivors of SAH^1^. In the current study, the effect of melatonin on mitophagy and the relationship between mitophagy and the NLRP3 inflammasome were investigated in early period of rat SAH model. We made the following observations: (1) treatment with melatonin upregulated mitophagy-associated proteins (PINK1/Parkin) and reduced mitochondrial damage and ROS generation after SAH; (2) administration of melatonin inhibited NLRP3 inflammasome activation after SAH and attenuated the inflammatory response; (3) melatonin treatment reduced neuronal cell death, brain edema and neurological dysfunction following SAH; and (4) pretreatment with 3-MA reversed the beneficial effect of melatonin on the inhibition of NLRP3 inflammasome activation. These findings suggested that melatonin upregulates mitophagy-associated proteins and suppresses ROS-induced NLRP3 inflammasome activation after SAH (Fig. 7).

The neuroinflammatory response contributes to the pathogenesis of post-SAH EBI with activated inflammatory cells and elevated pro-inflammatory cytokines^4^. Extensive studies have shown that either of inflammatory cells and pro-inflammatory cytokines was strongly associated with brain injury and neurological dysfunction after stroke^26–29^. Among the inflammatory responses after SAH, NLRP3 inflammasome activation has recently received attention. The NLRP3 inflammasome is activated by endogenous stimuli, including ROS and adenosine triphosphate (ATP)^9, 25^. Previous studies demonstrated that inhibition of the NLRP3 inflammasome exerted strong neuroprotective effects in the acute phase after SAH, which was related to reduction of pro-inflammatory cytokines^7^. Our study showed that melatonin strongly inhibits the inflammasome-mediated inflammatory response and microglia activation after SAH, which is consistent with previous studies^19–21^. However, the inner causality of NLPR3 response and microglia response was unclear in our study, which was worth of further investigation. Previous study demonstrated that ROS formation and oxidative stress promoted tissue inflammation and activated an immune response through NLRP3 inflammasome activation in some organs (brain, heart, kidney, and testis) after ischemia/reperfusion injury^24^. Thus, we hypothesized that the antioxidant effect of melatonin might be associated with its inhibition of NLRP3 inflammasome activation. Our study further suggested that upregulation of mitophagy-associated proteins and reduced ROS generation by melatonin treatment is linked to the inhibition of NLRP3 inflammasome activation. This study is the first to present a correlation between mitophagy and the NLRP3 inflammasome after SAH, and melatonin had a neuroprotective effect on this correlation.

Mitochondria are crucial for energy production and are sensitive to injury. In SAH-induced EBI, mitochondria play a critical role by the cessation of ATP synthesis and the overproduction of ROS as a result of sudden decreases in cerebral blood flow^30^. Inhibition of ROS-induced oxidative stress and inflammatory responses was beneficial for experimental SAH animals in our previous study^11^. The current study demonstrated that melatonin attenuates pathological changes in mitochondrial morphology and reduces ROS generation after SAH. Recently, the mitochondrion has been regarded as a center for signal transduction with involvement in autophagy^31^. In our previous study, we found that melatonin enhanced autophagy to protect against SAH-mediated EBI via inhibiting a mitochondrial apoptotic pathway^17^. Above all, this study revealed that melatonin upregulated mitophagy-associated proteins, eliminated damaged mitochondria and reduced ROS generation.

Mitophagy, as a selective form of autophagy, is chiefly responsible for clearance of damaged or dysfunctional mitochondria. The ubiquitin-like reaction shared by the general autophagy pathway and mitophagy employs the ubiquitin fold-containing protein complex Atg5-Atg12-Atg16 L1 and LC3^15^. Moreover, a recently identified specific mitophagy pathway showed that PINK1 and the E3 ubiquitin ligase Parkin accumulated on damaged mitochondria, promoted their segregation from the mitochondrial network, and targeted these organelles for autophagic degradation^15, 32, 33^. Our study suggested that melatonin up-regulated specific mitophagy markers as PINK1 and Parkin following SAH induction.

In the current study, we demonstrated that the neuroprotective effect of melatonin on post-SAH EBI was associated with mitophagy and NLPR3 inflammasome. However, there are several limitations to our study. An inhibitor of ROS scavengers should be applied to confirm the neuroprotective effect of melatonin following SAH. ROS-induced oxidative stress after SAH still warrants further investigation. The effects of different drugs administrated at different time points might have affected our results and should be more thoroughly investigated in further studies. Whether inflammasome activation affects the activity of mitophagy is still unclear. The causal relationship between mitophagy and reduced ROS generation should be confirmed by further experiments.

In conclusion, this study extended our understanding of the neuroprotective effects of melatonin on SAH-induced EBI and identified potential underlying mechanisms. We demonstrated that melatonin-upregulated mitophagy proteins played a protective role in post-SAH EBI. This neuroprotective effect was associated with the inhibition of NLRP3 inflammasome activation and reduced inflammatory responses.

## Materials and Methods

### Animal surgery and study design

Adult male Sprague–Dawley (SD) rats (310–330 g) were purchased from SLAC Laboratory Animal Co., Ltd. (Shanghai, China). Animals were maintained on a 12-hr light/dark cycle with controlled temperature and humidity. In total, 130 rats were randomly assigned to four groups: the sham group, the SAH + vehicle group, the SAH + melatonin (Mel) group, and the SAH + 3-methyladenine (3-MA) + Mel group. The SAH + Mel group was subjected to SAH and treated with melatonin; the SAH + 3-MA + Mel group was pretreated with 3-MA, subjected to SAH, and then treated with melatonin; the SAH + vehicle group was subjected to SAH and treated with vehicle; and the sham + vehicle group was subjected to a procedure similar to the SAH groups but without perforation and treated with vehicle. All endpoints in this study were investigated 24 h after SAH as in our previous studies. All experimental protocols were approved by the Institutional Animal Care and Use Committee of Zhejiang University and were specifically designed to minimize the number of animals used. Experiments with animals were carried out in accordance with the guidelines of the Animal Care and Use Committee of the Zhejiang University School of Medicine and the NIH Guide for the care and use of laboratory animals.

### The rat SAH model

The SAH model with endovascular perforation technique was prepared as previously described with modifications^17^. Briefly, after rats were anesthetized with pentobarbital (40 mg/kg, intraperitoneal injection), the left carotid artery and its branches were exposed. The external carotid artery was transected distally, and then a blunt 4-0 monofilament nylon suture was placed inside and advanced through the internal carotid artery until resistance was felt, which occurred at the junction of the middle cerebral artery and the anterior cerebral artery. The suture was further advanced approximately 3–5 mm to perforate the artery and create SAH with a stay for 10 s. A similar procedure was performed without perforation in the sham group. The mean arterial pressure, arterial pH, PO_2_, PCO_2_ and blood glucose levels were monitored during the operation, and no significant difference existed among the groups.

The severity of SAH was quantified according to a previously published grading scale^34^. The scale was based on the amount of subarachnoid blood in 6 segments of the basal cistern as follows: grade 0, no subarachnoid blood; grade 1, minimal subarachnoid blood; grade 2, moderate blood with visible arteries; and grade 3, blood clot covering all arteries within the segment. A total score ranging from 0 to 18 was obtained by adding scores from all 6 segments.

### Drug administration

Melatonin (N-acetyl-5-methoxytryptamine) (Sigma-Aldrich, St. Louis, MO, USA), dissolved in vehicle (1% ethanol in sterile saline) was injected intraperitoneally (5 ml/kg) in rats 2 h after SAH with a dosage of 150 mg/kg. The dose and the time point of melatonin injection were chosen based on previous studies^17, 19, 35^. Administration of vehicle (2 h after SAH) containing 1% ethanol had no measurable side effects. The autophagy inhibitor, 3-MA (Sigma-Aldrich, St. Louis, MO, USA), was dissolved in normal saline by heating the solution immediately before treatment. Rats were treated with 5-μl intracerebral ventricular injection of autophagy inhibitor 3-MA (400 nmol) or vehicle (saline) 20 min before SAH onset^36^.

### Evaluation of neurological deficits

Neurological deficits were evaluated 24 h after SAH according to the scoring system of Garcia *et al*.^22^. Briefly, the scoring system consists of six tests as follows: spontaneous activity, symmetry in the movement of four limbs, forepaw outstretching, climbing, body proprioception, and response to vibrissae touch. Each test was scored 0–3 or 1–3, and the total score ranged from 3 to 18. A lower score represents more serious neurological deficits. All the tests were assessed in a random blind manner.

### Brain water content measurement

Rats were sacrificed 24 h after SAH. The brain was removed and immediately weighed to obtain the wet weight (WW). The samples were dried at 105 °C for 24 h and weighed again to obtain the dry weight (DW). The brain water content was expressed as % brain water = [(WW − DW)/WW] × 100%^23^.

### ROS assay

The ROS assay was performed as previously described^11^. The left basal cortical sample was collected at 24 h after SAH, and total ROS levels were measured with ROS assay kit (Nanjing Jiancheng Bioengineering Institute, Nanjing, China) following the manufacturer’s instructions. Briefly, brain tissues were weighed and homogenized in 0.1 mM PBS, followed by centrifugation (1000 × *g*, 10 min, 4 °C). Protein concentration of the supernatant was determined using a DC protein assay kit (Bio-Rad, Hercules, CA, USA). The supernatant (190 µl) was added to 96 well plates and mixed with 1 mM DCFH-DA (10 µl). As a negative control, the supernatant was mixed with PBS. The samples were incubated at 37 °C for 30 min. The mixture was measured by spectrofluorophotometry at an excitation wavelength of 480 nm and an emission wavelength of 520 nm. The ROS levels in the brain tissue were expressed as fluorescence intensity/mg protein.

### Western blot analysis

Ipsilateral basal cortical samples facing the blood clots were extracted. Western blot was performed as described previously^37^. Briefly, cortical samples were homogenized, and centrifuged (1000 × *g*, 10 min, 4 °C). The supernatant was further centrifuged, and then, the protein concentration was determined using the DC protein assay kit (Bio-Rad, Hercules, CA, USA). An equal amount of protein (50 μg) was suspended in loading buffer, then denatured at 95 °C for 5 min, and loaded on an SDS-PAGE gel. After electrophoresis and transfer onto polyvinylidene fluoride membranes, the membranes were blocked with non-fat dry milk buffer for 2 h and then incubated overnight at 4 °C with primary antibodies for LC3 (2775, 1:1000, Cell Signaling), Atg5 (ab54033, 1:500, Abcam), Parkin (ab15954, 1:1000, Abcam), PINK1 (ab75487, 1:500, Abcam), NLRP3 (ab98151, 1:800, Abcam), ASC (ab155970, 1:1000, Abcam), caspase-1 (ab1872, 1:1000, Abcam), IL-1β (SC-23460, 1:500; Santa Cruz), IL-18 (ab71495, 1:1000; Abcam). The membranes were incubated with horseradish-peroxidase-conjugated secondary antibodies for 1 h at room temperature. The protein band densities were detected by X-ray film and quantified by ImageJ software (National Institutes of Health, Bethesda, MD, USA).

### Immunofluorescence staining and Fluoro-Jade C staining

Rats were sacrificed by intracardial perfusion of 0.1 mM PBS (pH 7.4) followed by 4% paraformaldehyde. Brains were immersed in 4% paraformaldehyde at 4 °C for 12 h for fixation and then immersed in a 30% sucrose solution for dehydration. Seven-micrometer-thick coronal frozen sections were collected. Immunofluorescence staining was performed as previously described^37^. The primary antibodies were anti-Beclin-1 (ab16998, 1:200, Abcam), anti-NeuN (MAB377, 1;500, Millipore), anti-GFAP (ab53554, 1:500; Abcam), Iba-1 (ab107159, 1:500; Abcam), with a rhodamine-conjugated secondary antibody (1:200, Jackson Immuno Research) and a fluorescein isothiocyanate-labeled secondary antibody (1:200, Jackson Immuno Research). The sections were rinsed, stained with DAPI (1 µg/ml, Roche Inc, Basel, Switzerland), rinsed again and mounted with glycerol. Fluorescence microscope (Olympus) was used to analyze the labeling.

For Fluoro-Jade C staining, specimens were dried for 30 min, rehydrated, and then incubated in 0.06% potassium permanganate solution for 10 min. After a water rinse, the slices were stained with 0.0001% Fluoro-Jade C solution for 5 min. Finally, the slices were dried again, cleared in xylene, and cover-slipped with mounting medium.

### TUNEL and PANT staining

TUNEL staining was designed to detect DNA double-strand damage according to the manufacturer’s protocol (Roche). A diaminobenzidine (DAB) substrate kit (Vector laboratories) was used for peroxidase staining, and the sections were then counter stained with hematoxylin. PANT staining was performed for the detection of cells with single-strand DNA damage. Slices were rinsed in 1% Triton-X-100 to permeabilize the sections and then 2% H_2_O_2_ to quench endogenous peroxidases. Afterwards, the sections were incubated with the PANT reaction mixture (10 mM 2-mercaptoethanol, 20 μg/ml BSA, 20 μM each of dGTP, dCTP and dTTP; 1 μM dATP, 19 μM biotinylated dATP and 40 units/ml of DNA polymerase I) at 37 °C for 90 min. Biotinylated dATP incorporated into damaged DNA was detected by incubation with streptavidin-horseradish peroxidase and DAB substrate.

The number of Iba-1-positive cells, Fluoro-Jade C-positive cells, TUNEL-positive cells and PANT-positive cells per field were counted by an investigator who was blinded to the experimental conditions. All of these cells were calculated randomly in five separate fields in four different slices/per animal.

### Transmission electron microscopy

Rats were sacrificed 24 h after SAH and perfused as described above. After the brain was exposed, 1 mm^3^ samples were collected from the ipsilateral basal cortex and then immersed in 2.5% glutaraldehyde overnight at 4 °C. The samples were rinsed in buffer and post-fixed with 1% osmium tetroxide for 1 h. After the samples were washed in distilled water 5 times, they were dehydrated in a graded ethanol series. The tissue was then infiltrated using a 1:1 mixture of propylene oxide and resin overnight. The samples were then embedded in resin. The sections were cut and stained with 4% uranyl acetate for 20 min and 0.5% lead citrate for 5 min. Ultrastructure was observed under a transmission electron microscope (Philiphs Tecnai 10).

### Statistical analysis

All data were expressed as the mean ± SEM and analyzed by GraphPad Prism (GraphPad Software Inc, San Diego, CA). One-way analysis of variance (ANOVA) followed by Tukey’s test was used for comparisons between groups. Neurological scores and SAH grade were analyzed with a one-way ANOVA on ranks, and then the Mann–Whitney U test was used for comparisons between groups. *P* < 0.05 was considered statistically significant.
